# A Rare Association of Congenital Glaucoma and Retinitis Pigmentosa: A 22-Year Follow-Up Case

**DOI:** 10.7759/cureus.105012

**Published:** 2026-03-10

**Authors:** Toshihiko Matsuo

**Affiliations:** 1 Ophthalmology, Graduate School of Interdisciplinary Science and Engineering in Health Systems, Okayama University, Okayama, JPN

**Keywords:** ciliopathy, cycloplegic refraction, full-correction glasses, goldmann perimetry, occlusion therapy, optical coherence tomography, photoreceptor ellipsoid zone, primary congenital glaucoma, retinitis pigmentosa, trabeculotomy

## Abstract

Primary congenital glaucoma is a rare congenital disease with a genetic background that shows high intraocular pressure due to ocular outflow anomalies. Retinitis pigmentosa is a predominant form of inherited retinal disorders. In this study, we present the case of a patient with primary congenital glaucoma in association with retinitis pigmentosa. A four-month-old female baby was brought to the emergency department due to corneal opacity in the left eye. The intraocular pressure measured by a hand-held applanation tonometer was 40 mmHg in the right eye and 36 mmHg in the left eye. She was otherwise healthy and diagnosed with primary congenital glaucoma. She immediately underwent trabeculotomy ab externo in both eyes under general anesthesia, and the intraocular pressure was lowered to 15 mmHg in the right eye and 12 mmHg in the left eye three weeks later. At the age of nine months, she was found to have retinal degeneration along the upper and lower vascular arcades of the fundus in both eyes and was diagnosed with retinitis pigmentosa. At the age of one year and 10 months, the visual acuity was measured at 0.2 in the right eye and 0.2 in the left eye for the first time by a preferential looking procedure. The intraocular pressure was 9 mmHg in both eyes under sedation, and she did not use any topical medication. At the age of three years and three months, the uncorrected visual acuity and best-corrected visual acuity with myopic astigmatism correction were 0.1 and 0.15, respectively, in the right eye and 0.6 and 0.7, respectively, in the left eye. Occlusion therapy with an eye patch over the left eye for one hour daily was started. At the age of four years and 10 months, the best-corrected visual acuity was 0.7 in both eyes. At the age of six years, occlusion therapy was discontinued, and full-correction glasses were prescribed, based on cycloplegic refraction. The visual acuity in the right eye decreased to 0.3 at the age of 11 years and further to 0.1 at the age of 12 years, while the visual acuity in the left eye remained 0.8. Afterwards, she maintained a visual acuity of 0.1 in the right eye and 0.8 in the left eye until the age of 22 years. An incidental presence of primary congenital glaucoma in this patient led to the detection of retinitis pigmentosa in earlier years and allowed long-term follow-up for 22 years. Even though genetic testing was not performed for this patient, the abnormal function of primary cilia, designated as ciliopathy, might explain the co-occurrence of primary congenital glaucoma and retinitis pigmentosa.

## Introduction

Congenital disorders of the eye are largely classified into anomalies that involve the anterior part of the eyeball, designated as anterior segments, and the posterior part of the eye, as posterior segments, including the retina and optic nerve [1]. Primary congenital glaucoma [2] is a part of anterior segment dysgenesis that indicates congenital ocular anomalies involving the anterior part of the eyeball, such as the cornea, iris, and trabecular meshwork at the iridocorneal angle [3,4]. In terminology, primary congenital glaucoma is used for isolated, non-syndromic situations that occur before the age of three years. Later development of glaucoma is designated as developmental or juvenile glaucoma. Syndromic early-onset glaucoma, such as glaucoma in Axenfeld-Rieger syndrome, is called secondary developmental glaucoma. In congenital glaucoma, including developmental glaucoma, aqueous humor outflow from the anterior chamber to the Schlemm canal, which connects to venous channels, is blocked by anomalous development of the trabecular meshwork to varying extents [5-7].

Babies with congenital glaucoma present corneal opacity with edema as an early sign, and consequently, epiphora and photophobia as symptoms. Later in the course, the eye with congenital glaucoma becomes enlarged as a sequel to high intraocular pressure in the setting of soft scleral tissue, referred to as buphthalmos. Early surgical intervention is recommended to lower the intraocular pressure to save the optic nerve fibers, usually by trabeculotomy ab externo, referring to trabeculotomy from outside of the eye [8-10].

Retinitis pigmentosa is the most prevalent inherited retinal disorder and presents visual field constriction and night blindness as symptoms [11]. The visual field constriction often manifests as concentric narrowing in both eyes and is caused by the different extent of involvement with retinal dystrophy. The disease progression, which is the speed of spatial and temporal extension of retinal dystrophy, varies largely from individual to individual. Retinitis pigmentosa, which occurs in association with other systemic diseases, is designated as syndromic retinitis pigmentosa [12,13]. So far, congenital glaucoma has not been described in association with retinitis pigmentosa. Here, we report the case of a patient who underwent trabeculotomy in both eyes with primary congenital glaucoma at the age of four months. She was found to have retinitis pigmentosa six months later and was followed until the age of 22 years. This association of primary congenital glaucoma and retinitis pigmentosa has not been previously reported to my knowledge.

## Case presentation

A four-month-old female baby was brought to the emergency department because the parents noticed a cloudy cornea in the left eye. The intraocular pressure measured by a hand-held applanation tonometer was 40 mmHg in the right eye and 36 mmHg in the left eye. She was diagnosed with primary congenital glaucoma and referred to a university hospital. She had been delivered as a first child at the gestational age of 39 weeks with a birthweight of 3,096 g with no complications. She was healthy, and nothing was pointed out during a checkup at three months of age. There was no consanguinity or family history of congenital glaucoma or retinitis pigmentosa. The next day at the referral, the intraocular pressure was 33 mmHg in the right eye and 28 mmHg in the left eye. The cornea in the right eye was clear and allowed fundus visualization to observe the normal-colored optic disc with a cup-to-disc ratio of 0.6. The cornea in the left eye was opaque to prevent the fundus from being visualized. Systemic examination by a pediatrician was normal, and she underwent trabeculotomy ab externo in both eyes under general anesthesia. At the time of surgery, the corneal diameter in vertical and horizontal dimensions was 12 × 13 mm in the right eye and 14 × 13 mm in the left eye, indicating the enlargement of the left cornea. The gonioscopic observation was hindered by corneal opacity, and corneal pachymetry was not performed.

Three weeks after the surgery, the intraocular pressure was 15 mmHg in the right eye and 12 mmHg in the left eye. The cornea in the right eye was clear, and the cornea in the left eye showed the remaining circular opacity in the upper part. The anterior chamber was deep, and the lens was clear in both eyes. The optic discs in both eyes were normal-colored with a cup-to-disc ratio of 0.4. Postoperative topical 0.5% levofloxacin and 0.1% betamethasone eyedrops were discontinued. A month later, in cycloplegic refraction with topical 1% cyclopentolate, the patient showed moderate myopic astigmatism in both eyes: spherical -3.75 diopters and cylindrical -1.5 diopters at the axis of 45 degrees in the right eye and spherical -4.75 diopters and cylindrical -1.5 diopters at the axis of 70 degrees by hand-held refractometry. The pupillary dilatation with topical 1% cyclopentolate was poor at 5 mm in diameter in the right eye and 4 mm in the left eye. Her eye position was orthophoric, and glasses were not prescribed. The intraocular pressure was 9 mmHg in both eyes with no use of topical medication. She was free from symptoms such as epiphora, photophobia, and blepharospasm.

**Figure 1:**
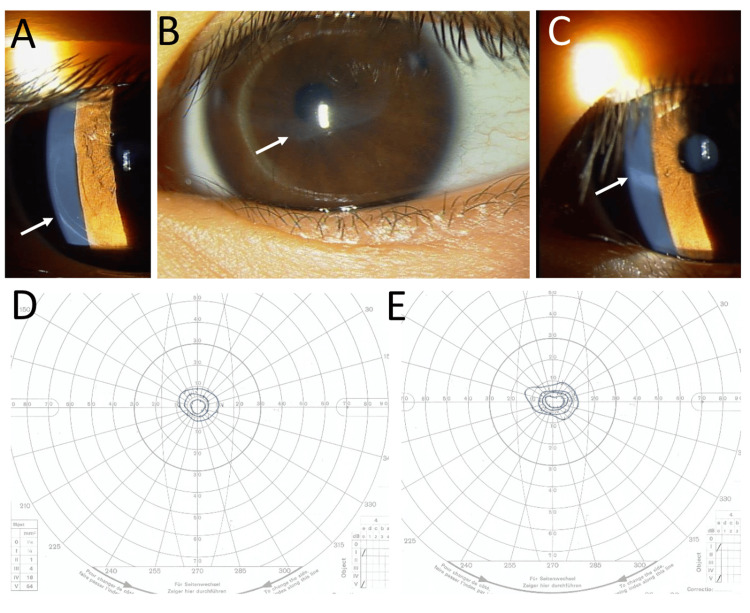
Slit-lamp photographs at the age of five years and Goldmann perimetry at the age of seven years. Slit-lamp photographs at the age of five years, showing track-like Descemet membrane rupture of the cornea in the right eye (arrow, A) and residual corneal opacity (arrows, B, C) in the left eye. Goldmann perimetry for the first time at the age of seven years, showing concentric constriction to 10 degrees meridian in the right eye (D) and left eye (E). Outermost to inner isopters in perimetry are sequentially V/4, I/4, I/3, I/2, and I/1.

At the age of nine months, she was found to have retinal degeneration along the upper and lower vascular arcades of the fundus in both eyes and was diagnosed with retinitis pigmentosa. At the age of one year and 10 months, the visual acuity was measured to be 0.2 in the right eye and 0.2 in the left eye, separately in each eye for the first time, by a preferential looking procedure (Table 1). The intraocular pressure was 9 mmHg in both eyes under sedation, and she did not use any topical medication. At the age of two years and seven months, the visual acuity without correction was 0.15 in the right eye and 0.4 in the left eye by the measurement with Landolt-C cards. No refractive error was detected by cycloplegic refraction. At the age of three years and three months, the uncorrected and best-corrected visual acuity was 0.1 and 0.15, respectively, with cylindrical -1.75 diopters at the axis of 180 degrees in the right eye and 0.6 and 0.7, respectively, with cylindrical -2.5 diopters at the axis of 180 degrees in the left eye. Occlusion therapy with an eye patch over the left eye for one hour daily was started. At the age of four years and 10 months, the best-corrected visual acuity was 0.7 in both eyes. She showed a track-like Descemet membrane rupture in the right eye (Figure 1A) and residual corneal opacity in the left eye (Figures 1B, 1C). At the age of six years, the occlusion therapy was discontinued, and full-correction glasses were prescribed, based on cycloplegic refraction. Goldmann perimetry for the first time at the age of seven years showed concentric constriction of visual fields within 10 degrees meridian in both eyes (Figures 1D, 1E). Electroretinograms in both eyes showed flat, distinguished responses. The axial length measurements were not performed in the course as the refractive errors could be measured by a table-fixed autorefractor.

**Table 1:** Visual acuity and refractive errors in follow-up of 22 years. n.c.: not correctable; s.: spherical; c.: cylindrical; D: diopter; Ax: axis

Age	Uncorrected visual acuity (best-corrected visual acuity with refractive errors) in decimals		Features
	Right eye	Left eye	
9 months	0.005	0.005	Preferential looking, 0.2 with both eyes open
1 year 10 months	0.2	0.2	Preferential looking
2 years 7 months	0.15	0.4	Landolt-C cards
3 years	0.15	0.5	Landolt-C cards
3 years 3 months	0.1 (0.15 × c.-1.75D Ax180)	0.6 (0.7 × c.-2.5D Ax180)	Landolt-C chart, occlusion with eye patch over left eye for 1 hour daily
4 years	0.2 (0.4 × s.-1.5D and c.-1.5D Ax180)	0.6 (n.c.)	Landolt-C chart
4 years 10 months	0.2 (0.7 × s.-1.5D and c.-1.0D Ax180)	0.5 (0.7 × c.-2.5D Ax180)	Landolt-C chart
6 years	0.2 (0.7 × s.-1.5D and c.-1.0D Ax180)	0.5 (0.7 × s.+1.5D and c.-3.0D Ax180)	Landolt-C chart, eye patch discontinued, glasses prescription based on cycloplegic refraction
10 years	0.2 (0.6 × s.-1.25D and c.-1.5D Ax180)	0.5 (0.8 × s.+0.75D and c.-2.5D Ax180)	Landolt-C chart, glasses changed
11 years	0.2 (0.3 × s.-1.25D and c.-1.5D Ax180)	0.5 (0.8 × s.+0.75D and c.-2.5D Ax180)	Landolt-C chart, glasses occasionally used
12 years	0.1 (0.1 × s.-1.25D and c.-1.5D Ax180)	0.5 (0.8 × s.+0.75D and c.-2.5D Ax180)	Landolt-C chart
15 years	0.1 (0.1 × s.-1.25D and c.-1.5D Ax180)	0.5 (0.8 × s.+0.75D and c.-2.5D Ax180)	Landolt-C chart, glasses not used anymore
18 years	0.1 (0.1 × s.-3.75D and c.-1.0D Ax180)	0.5 (0.8 × s.-1.25D and c.-1.5D Ax180)	Landolt-C chart
22 years	0.1 (0.1 × s.-5.0D and c.-1.0D Ax180)	0.5 (0.8 × s.-1.25D and c.-1.5D Ax180)	Landolt-C chart

**Figure 2:**
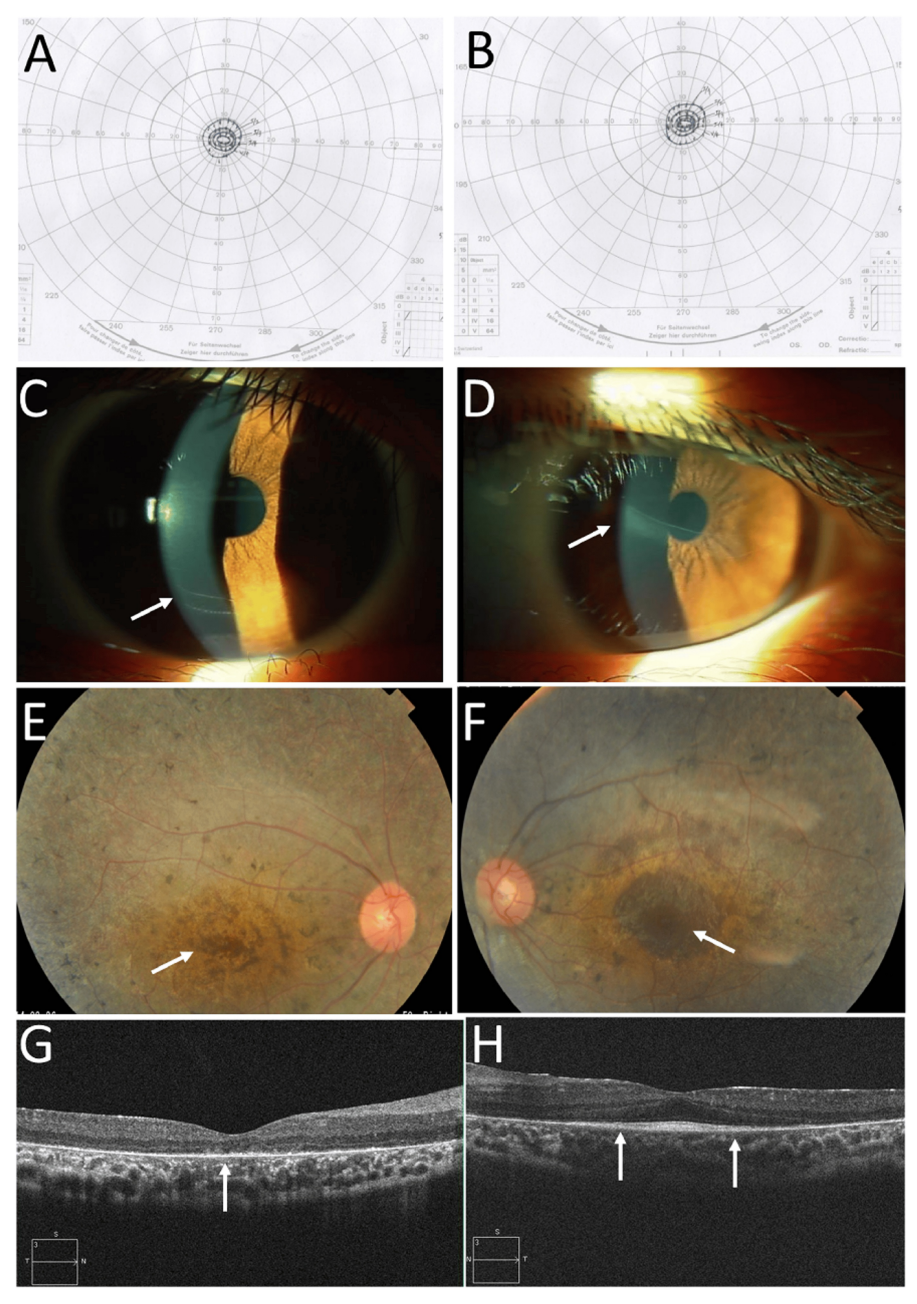
Goldmann perimetry at the age of nine years and ophthalmic images at the age of 10 years. Goldmann perimetry at the age of nine years, showing the same level of concentric constriction in right eye (A) and left eye (B). Outermost to inner isopters in perimetry are sequentially V/4, I/4, I/3, I/2, and I/1. Slit-lamp photographs at the age of 10 years, showing deep anterior chamber in both eyes (C: right eye, D: left eye) with track-like Descemet membrane rupture of the cornea (arrows). Fundus photographs (E, F) and horizontal section images of optical coherence tomography (G, H) in right eye (E, G) and left eye (F, H) at the age of 10 years, showing normal-colored optic discs and preserved retina in central small areas (arrows, E, F). Note the absence of photoreceptor ellipsoid zone in the right eye (arrow, G) and preserved photoreceptor ellipsoid zone between arrows in the left eye (H).

She maintained the visual acuity of 0.7 in both eyes until the age of 10 years. The visual fields in both eyes remained the same (Figures 2A, 2B). The visual acuity in the right eye decreased to 0.3 at the age of 11 years and further to 0.1 at the age of 12 years, while the visual acuity remained 0.8 in the left eye. She showed the same track-like Descemet membrane rupture in both eyes (Figures 2C, 2D) and entire retinal degeneration except for a small area of the macular center in both eyes (Figures 2E, 2F). Optical coherence tomography revealed the loss of the photoreceptor ellipsoid zone in the right eye (Figure 2G) and a narrow area of the preserved ellipsoid zone in the left eye (Figure 2H).

**Figure 3:**
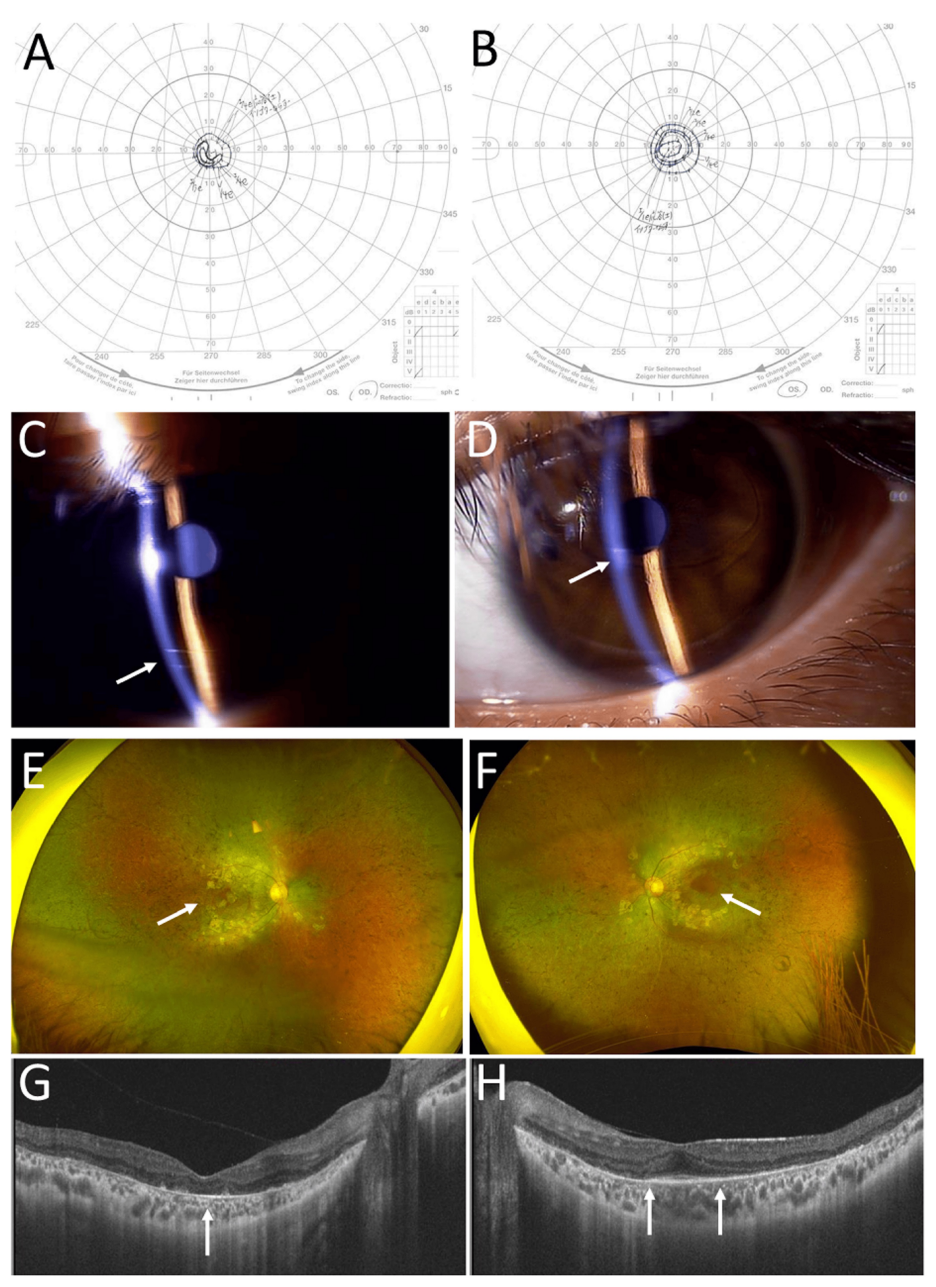
Goldmann perimetry and ophthalmic images at the age of 22 years. Goldmann perimetry at the age of 22 years, showing the same level of concentric constriction in the right eye (A) and left eye (B). Outermost to inner isopters in perimetry are sequentially V/4, I/4, I/3, I/2, and I/1. Note partial loss of inner isopters in the right eye (A). Slit-lamp photographs at the age of 22 years, showing deep anterior chamber in both eyes (C: right eye, D: left eye) with track-like Descemet membrane rupture of the cornea (arrows). Wide-field fundus photographs (E, F) and horizontal section images of optical coherence tomography (G, H) in right eye (E, G) and left eye (F, H) at age 22 years, showing normal-colored optic discs and preserved retina in small central areas (arrows, E, F). Note the absence of photoreceptor ellipsoid zone in the right eye (arrow, G) and narrowing and obscuring of preserved photoreceptor ellipsoid zone between arrows in the left eye (H).

Afterwards, she maintained the visual acuity of 0.1 in the right eye and 0.8 in the left eye until the latest visit at the age of 22 years (Table 1). The intraocular pressure was 10 mmHg in both eyes. The visual field in the right eye showed depression of inner isopters (Figure 3A) by Goldmann perimetry, while the visual field in the left eye remained the same (Figure 3B). The cornea in both eyes was clear except for track-like Descemet membrane ruptures (Figures 3C, 3D), and the entire retina showed degeneration in both eyes (Figures 3E, 3F). Optical coherence tomography showed no photoreceptor ellipsoid zone in the right eye (Figure 3G) and a further narrowed area of the ellipsoid zone at the center (Figure 3H), compared with the age of 10 years (Figure 2H). She had night blindness but had not experienced any difficulty in studying at school. Otherwise, she was free from any symptoms and did not use any medication. She and her family did not wish to undergo genetic testing.

## Discussion

This patient with retinitis pigmentosa was unique in the presentation with primary congenital glaucoma in both eyes at the age of four months. After the intraocular pressure was successfully lowered by an early surgery with trabeculotomy in both eyes, she was found to have retinitis pigmentosa at the age of nine months. She did not use any topical medication for glaucoma in the follow-up period of 22 years. The early diagnosis of retinitis pigmentosa before the age of one year is rare in the usual clinical course of the disease. The presence of congenital glaucoma in this patient allowed us to observe how retinitis pigmentosa would be stable and to what extent the disease would show deterioration in the course of 22 years. She might not have been diagnosed with retinitis pigmentosa in the earlier age, as she did not have any problems in daily life except for night blindness.

In Japan, trabeculotomy ab externo, in place of goniotomy, has been the standard surgical procedure for congenital glaucoma for more than 40 years since the 1980s [8]. Because the gonioscopic visualization is usually hindered by corneal opacity in primary congenital glaucoma, gonioscopy-assisted transluminal trabeculotomy cannot be performed as can be done in developmental glaucoma. Furthermore, the anomalous positioning of the Schlemm canal, which is often found in primary congenital glaucoma, is not suitable for the surgical approach from inside the anterior chamber [10]. This patient was indeed lucky to achieve the reduction of the intraocular pressure in both eyes by the one-time surgery. She maintained normal levels of the intraocular pressure in both eyes in the long-term course of 22 years and was free from any symptoms such as photophobia and epiphora.

Refractive error correction with glasses is crucial for the development of visual acuity to avoid amblyopia in pediatric eye diseases, including congenital anomalies and retinopathy of prematurity [1,14]. Hence, cycloplegic refraction with 1% cyclopentolate instillation was performed at the age of five months, and visual acuity testing with the preferential looking procedure was performed at the age of nine months for this patient, as summarized in Table 1. In these detailed ophthalmic evaluations, the patient was found to have retinitis pigmentosa. At the age of three years, the patient showed a poor level of visual acuity in the right eye, compared to the left eye, and occlusion therapy with an eyepatch over the left eye was started for one hour daily. The patient gained the same level of visual acuity in the right eye as the left eye at the age of four years. The occlusion therapy was discontinued, and full-correction glasses were prescribed at the age of six years when she was enrolled in an elementary school. She did not experience any problem in studying at the school. In retrospect, it would be surprising for the patient to gain the same visual acuity of 0.7 in the right eye as the left eye with the background of no photoreceptor ellipsoid zone at the center in the right eye at the age of 10 years (Figure 2G). She might have had a limited area of the remaining photoreceptor ellipsoid zone in the right eye at the age of three to four years, when optical coherence tomography was not available.

She showed a slowly progressive decline in visual acuity in the right eye from the age of 10 years, resulting in a visual acuity of 0.1 at the age of 12 years (Table 1). Optical coherence tomography showed apparent loss of the photoreceptor ellipsoid zone in the right eye, compared with the maintenance of the ellipsoid zone at the center in the left eye at the age of 10 years (Figure 2G, 2H). She maintained the visual acuity of 0.1 in the right eye and 0.8 in the left eye for 12 years until the latest visit at the age of 22 years. Optical coherence tomography at the age of 22 years showed that the preserved area of the photoreceptor ellipsoid zone in the left eye was further narrowed (Figure 3H) in comparison with that at the age of 10 years (Figure 2H). In parallel, Goldmann perimetry showed that the central visual field in the left eye (Figure 3B) was maintained almost at the same level as that noted at 12 years previously (Figure 2B).

To my knowledge, there has been no report on the association of retinitis pigmentosa with primary congenital glaucoma. It is well-known that patients with retinitis pigmentosa often develop angle-closure glaucoma [15,16]. The association with angle-closure glaucoma did not fit with the present patient. In the present patient, no consanguinity in the family and the absence of family history of congenital glaucoma or retinitis pigmentosa suggest a recessive trait for both conditions. At present, multiple disease-causing genes have been identified in congenital glaucoma [2,17]. Among these genes, cytochrome P450 family 1 subfamily B member 1 (*CYP1B1*) and latent transforming growth factor beta binding protein 2 (*LTBP2*) are the most well-known genes that cause autosomal recessive congenital glaucoma [17]. In addition, multiple genes that are associated with anterior segment dysgenesis would be involved in the development of congenital glaucoma [18].

These genes for congenital glaucoma and anterior segment dysgenesis are not found in the list of genes for retinitis pigmentosa [11,12]. A connecting line that might link congenital glaucoma with retinitis pigmentosa is a disease framework of ciliopathy [19]. The photoreceptors have primary cilia called connecting cilia, which join the outer segment with the inner segment of the cell body. Most diseases in the entity of syndromic retinitis pigmentosa, such as Usher syndrome, Bardet-Biedl syndrome, and Senior-Loken syndrome, are characterized as ciliopathies, which indicate the abnormal function of the primary cilia as an underlying cause [12,13]. The trabecular cells in the trabecular meshwork, which constitute the outflow pathway of the aqueous humor, also have the primary cilia [19]. Meanwhile, neural crest cells play a major role in the development of the anterior segment of the eye and would be involved in the anterior segment dysgenesis [20,21]. Under the circumstances, there have been lines of evidence to suggest that anterior segment dysgenesis might also be designated as ciliopathy [22-24].

Otherwise, the co-occurrence of congenital glaucoma and retinitis pigmentosa might be by chance in the present patient. The prevalence of primary congenital glaucoma is up to 1 in 10,000 to 20,000 people [2], while the prevalence of retinitis pigmentosa is up to 1 in 3,000 to 4,000 people [11,12]. In a quite rarer situation, this patient might harbor two different recessive traits for congenital glaucoma and retinitis pigmentosa. The lack of genetic analysis is a major limitation of this study. The possible alternative mechanisms might be de novo mutations or digenic inheritance. Whole-exome sequencing in such cases could potentially identify a novel genetic link.

## Conclusions

The present patient showed a rare association of retinitis pigmentosa with primary congenital glaucoma. The patient presented with corneal opacity in the left eye and was diagnosed with primary congenital glaucoma at the age of four months. She underwent an early trabeculotomy in both eyes and gained successful lowering of the intraocular pressure without topical medication for a long time. During detailed ophthalmic evaluation after the surgery, she was found to have retinitis pigmentosa at the age of nine months and was followed until the age of 22 years. The presentation with primary congenital glaucoma at the first visit might have led to an incidental early diagnosis of retinitis pigmentosa in the course of this patient. The present study highlights the value of comprehensive ophthalmic evaluation in all children presenting with congenital glaucoma. Even though genetic testing was not performed for this patient, the abnormal function of primary cilia, which is designated as ciliopathy, might explain the co-presence of primary congenital glaucoma and retinitis pigmentosa.
